# Microbiomes of colored dental biofilms in children with or without severe caries experience

**DOI:** 10.1002/cre2.317

**Published:** 2020-08-06

**Authors:** Nobuko Nagai, Hiromi Homma, Atsuo Sakurai, Naoko Takahashi, Seikou Shintani

**Affiliations:** ^1^ Department of Pediatric Dentistry Tokyo Dental College Tokyo Japan

**Keywords:** dental education, periodontal disease, prevention

## Abstract

**Background:**

Biofilm coloration can compromise maturation and increase the risk of oral disease in adulthood, though children with colored biofilm do not always demonstrate a poor oral health status.

**Aim:**

The microbial compositions of colored and white biofilms in children were compared.

**Design:**

Thirty‐two dental biofilm samples from 16 children (age < 13 years) were analyzed using 16S rRNA pyrosequencing, then the subjects were divided into severe caries and healthy (caries‐free) groups. Correlations between microbiomes and oral health status were also examined.

**Results:**

Phylogenetic analysis revealed no distinctly different patterns between colored and white biofilms. In the severe caries group, genus *Actinomyces*, *Cardiobacterium*, *Kingella*, *Lautropia*, and *Veillonella*, and family *Neisseriaceae* were detected, though abundance was significantly different between colored and white biofilm specimens, in contrast to the healthy group. In addition, five colored biofilm samples from the severe caries group contained greater than 15% *Actinomyces*, which led us to consider that genus to be possibly associated with formation of colored biofilm in children.

**Conclusions:**

Our findings indicate that differences in bacterial composition between colored and white biofilms are higher in individuals with severe caries. Additional research may reveal the significance of colored dental biofilm in children.

## 1 INTRODUCTION

Formation of dental biofilm, also referred to as plaque, begins with adsorption of the pellicle on the tooth surface. In the initial phase of biofilm formation, the initial bacteria to attach to the pellicle are mainly Gram‐positive cocci, such as streptococci, followed by Gram‐positive bacilli and then obligatory anaerobic Gram‐negative bacteria (Kolenbrander et al., 2006). When brushing is not promptly performed, biofilm develops and gradually becomes thick, with mature biofilm known to be responsible for oral diseases such as dental caries and periodontal disease (Socransky & Haffajee, 2005; Zijnge, Van Leeuwen, Degener, et al., 2010).

Dental biofilm is transparent or white in the early stages, though can become yellow or gray in color as it matures, and is termed colored biofilm. In children, the color is white in most cases, though can occasionally be yellow or yellow‐brown (Yoshimura, Suzuki, Nakaoka, et al., 2008). Mature biofilm in adults is rich in bacteria, in particular, red or orange complex species, which have been shown to be strongly correlated with periodontal disease (Teles, Teles, Frias‐Lopez, Paster, & Haffajee, 2013). On the other hand, a report of child cases in which dental caries and gingivitis were absent, noted that oral health status was not poor despite the presence of colored biofilm (Yoshimura et al., 2008).

Previous reports have found that differences in biofilm color reflect not only extrinsic staining but also changes in the bacterial composition of the oral microbiome (Li et al., 2015). *Porphyromonas gingivalis* and *Aggregatibacter actinomycetemcomitans* require bivalent iron ions for their growth, while the presence of oxidized trivalent iron ions leads to brown or black precipitation (Nakayama et al., 1998; Rhodes, Shoemaker, Menke, Edelmann, & Actis, 2007). Thus, substances needed for bacterial growth and their metabolites are also considered to be involved in biofilm coloration. However, little is known regarding the bacterial composition of colored as compared to white or colorless biofilm (hereinafter termed “white” biofilm) in children. In the present study, biofilms were obtained from child subjects and analyzed using 16 s rRNA gene pyrosequencing with a next‐generation sequencer as well as conventional polymerase chain reaction (PCR) assays. Results showing the bacterial compositions of colored and white biofilms collected from the same subjects were compared. In addition, we analyzed the association of dental caries prevalence with bacterial composition in biofilm samples obtained from the present subjects.

## 2 MATERIALS AND METHODS

### 2.1 Inclusion criteria and sampling

This study was conducted after receiving approval from our institution's ethics committee (No. 251). Prior to sampling, the parents/guardians or caregiver of each child received an explanation regarding the study design, and written consent for participation was obtained. Of children who visited the Pediatric Dental Clinic at our hospital, those aged less than 13 years and possessing colored biofilm on the buccal side of the upper first or upper primary second molar were considered eligible (Table 1). To compare findings between good and poor oral status, subjects were classified into the severe caries (10 or more deciduous teeth with dental caries, or with caries experience) and healthy (absence of caries experience, with either deciduous or permanent teeth) groups. Children whose primary teeth had already been replaced were excluded. In addition, children suffering from a common cold at the time of sampling, with a systematic disease, or who had taken antimicrobial drugs within 1 month prior to sampling were excluded. The total number of subjects enrolled was 32.

**TABLE 1:** Demographic and clinical characteristics of subjects

	No. of subjects	Mean age, years (range)	*df*, %	DMF, %	With dental decay, %	With caries experience, %
Conventional PCR	32	8.6 (1–12)	41.2	9.2	31.1	68.9
Severe caries	20	7.6	73.6	11.6	40	100
Healthy	12	9.3	0	0	0	0
16S rRNA pyrosequencing	16	8.2 (6–10)	34.4	2.9	31.3	56.3
Severe caries	9	8.2 (7–9)	61	5	56	100
Healthy	7	8.1 (6–10)	0.0	0.0	0	0

The presence or absence of colored biofilm in each child was visually confirmed by two experienced pediatric dentists using a headlight with the subject seated in a dental chair (Figure S1a) and a color scale bar (Figure S1b). The number of primary or permanent teeth with caries experience, or presence of dental decay was also determined from intraoral examination findings as well as a review of past clinical records.

Using a sterile dental excavator, supragingival biofilm on the buccal side of the upper first or upper primary second molar was collected. In addition, white biofilm from the site closest to the colored biofilm site (buccal side of adjacent tooth or opposite upper molar) was also collected from each child. Each of the samples was immediately immersed in 2% glycerol‐containing TE buffer (10 mM Tris–HCl, 1 mM EDTA; pH 8.0), then washed twice with 1 ml of TE buffer and stored at −80°C until analysis (Xu et al., 2014).

### 2.2 DNA extraction

DNA was extracted using a MORA‐EXTRACT kit (Kyokuto Pharmaceutical Industrial, Tokyo Japan), according to the manufacturer's protocol. Included in the kit were 2‐ml centrifuge tubes containing lysis buffer and zirconia beads, which were used to efficiently crush the firm cell walls of gram positive bacteria. DNA concentration and purity level were determined with a Nano drop 8000 spectrophotometer (Thermo Fisher Scientific, Waltham, MA).

### 2.3 Conventional PCR

To detect specific bacteria in each sample, PCR was performed using a previously reported method (Ashimotoa, Bakker, & Slots, 1996; Conrads et al., 1996; Goncharoff, Figurski, Stevens, & Fine, 1993; Hoshino et al., 2004; Kobayashi et al., 2008; Mättö, Saarela, Alaluusua, & Oja, 1998). Eleven different bacteria known to be contained in dental biofilm, and considered to be involved in the onset of dental caries and periodontal disease were examined (Table S1).

### 2.4 16S rRNA gene pyrosequencing

Analysis with a next‐generation sequencer was performed using a total of 32 samples (including both colored and white biofilms) collected from 16 of the subjects. Of those 16 subjects, 7 were allocated to the healthy and 9 to the severe caries group. An amplicon library of the 16S rRNA gene V3‐V4 hypervariable region was prepared from bacterial genomic DNA using an adaptor sequence‐added universal primer and TaKaRa Taq HS Low DNA (Takara Bio, Kusatsu, Japan). Amplification was conducted under the following cycling conditions: 30 cycles of 30 s of denaturation at 94°C, 40 s of annealing at 55°C, and 40 s of extension at 72°C, followed by a 7‐min final extension at 72°C. The length of the amplified product was confirmed with 1.5% agarose gel electrophoresis, followed by purification using a commercially available kit (NucleoSpin Gel and PCR clean up, MACGEREY‐NAGEL, Düren, Germany). Then, using a Nextera XT Index kit (Illumina, San Diego, CA), each sample was added to a unique index sequence, followed by a second purification with Agencourt AMPure XP (Beckman Coulter, Krefeld, Germany). The library obtained was subjected to concentration measurement with a Quantus Fluorometer (Promega, Madison, WI), with a quality check performed with an Agilent Bioanalyzer 2100 (Agilent Technologies, Santa Clara, CA). Thereafter, equal amounts of all libraries were combined and sequenced using the Illumina MiSeq sequencing platform (Illumina) and a MiSeq Reagent Kit, v.3 (Illumina).

### 2.5 Sequence data processing and statistical analysis

All results obtained from sequencing were analyzed using the Bioinformatics pipeline QIIME (ver. 1.9.1; Caporaso et al., 2010). First, data were allocated to individual samples according to index sequences. Then, on the basis of the sequence of overlapping regions, forward and reverse paired‐end sequence reads were combined, with 20% or lower mismatches deemed to be acceptable. Using the default setting of QIIME, reads with too few bases or containing low‐quality bases were excluded. Subsequently, chimeric sequences were detected using the USEARCH 64‐bit software package (ver. 6.1.544; Edgar & Flyvbjerg, 2014). Finally, index and primer sequences of individual reads were removed. Sequence data thus obtained were deposited in the DNA Data Bank of Japan (DDBJ) Sequence Read Archive (accession no. DRA007072).

Following a quality check, sequence reads were classified into operational taxonomy units (OTUs), with OTUs with 99% or greater homology counted as a single cluster (Edgar, 2010). Next, reads matching the reference sequence in the SILVA database (release 119) were explored to estimate bacterial taxa at a class as low as possible (Quast et al., 2013). OTUs detected only once per sample were excluded from further analysis. To examine the richness and alpha diversity of the microbiomes, observed OTUs and Shannon index values were calculated. For the bacterial taxa observed in all samples, a heat map was prepared to show relative abundance. Unweighted and weighted UniFrac distances, and Spearman's rank correlation coefficient (SCC) were calculated to determine beta diversity. Based on weighted UniFrac distance metrics, phylogenetic trees and principal coordinate analysis (PCoA) were used to compare colored and white biofilms (Shi, Qin, Chen, & Xia, 2016). Furthermore, that comparison was also made within each of the healthy and severe caries groups. Statistical analyses were performed using Student's *t*‐test, a Chi‐square test, and Wilcoxon's test with JMP software (ver. 10.0.2; SAS Institute Inc., NC).

## 3 RESULTS

Consent for participation was obtained for subjects found to have colored biofilm on the upper first or upper second deciduous molar (n = 32; Table 1). The youngest at the time of such detection was 1 year 2 months old, while the mean age of all was 8.2 years. Conventional PCR analysis of biofilm samples obtained from all subjects was performed. In addition, 16S rRNA gene pyrosequencing was done using 32 samples from 16 subjects aged 6–10 years who fulfilled the definition of the healthy or severe caries group, as described in the Materials and Methods section (Table 1). After performing a quality check, the total number of reads from the samples was 3,013,105, while the mean number of reads was 94,159 (range 18,284–291,327; Table S2). Furthermore, the number of observed OTUs with 99% similarity from those 32 samples was 587, with an average of 145 per sample (range 83–236).

**FIGURE 1:**
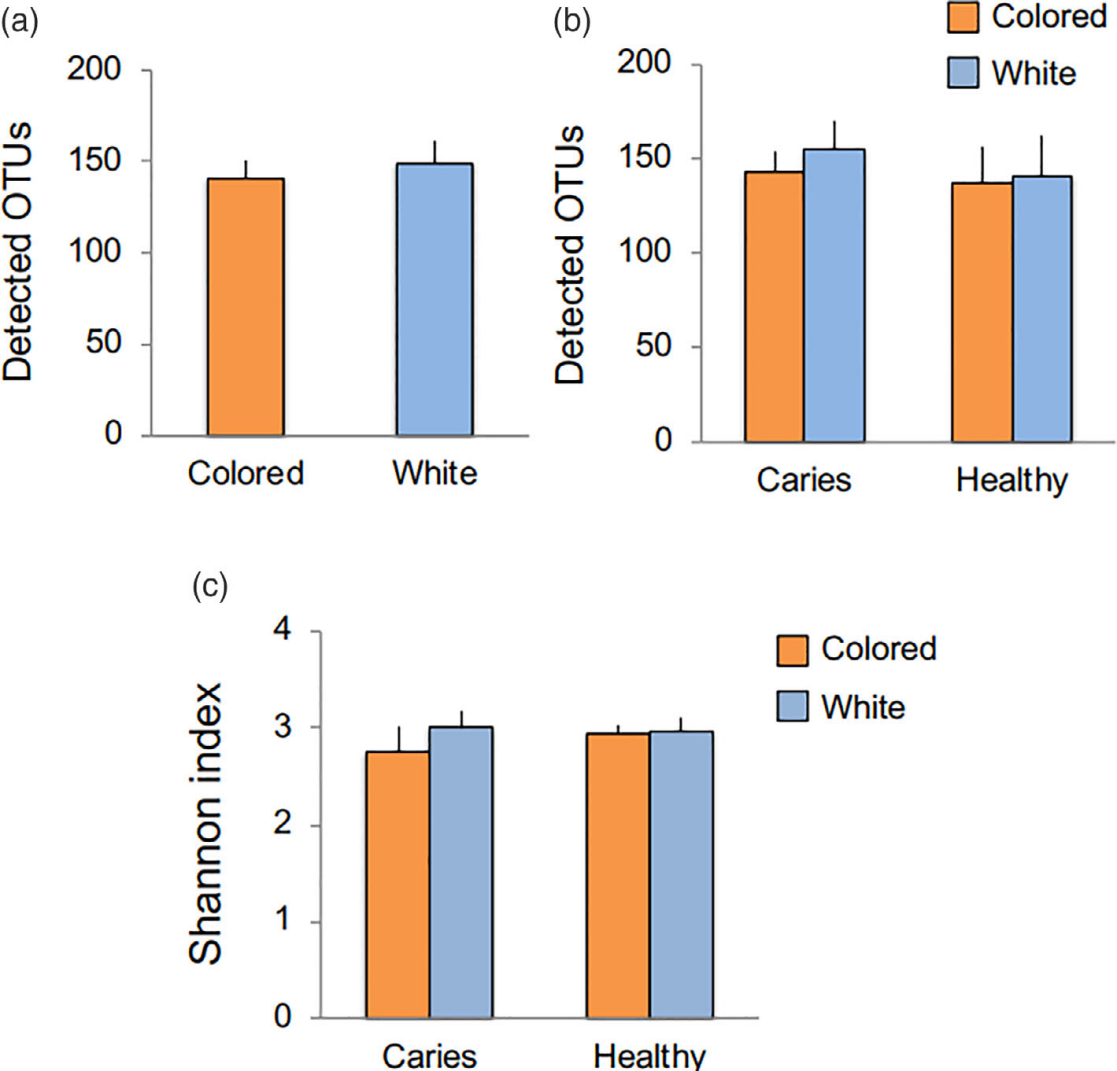
Microbial richness and diversity based on observed OTUs and Shannon index value. (a) Average number of OTUs in all colored and white biofilm specimens. (b) Average number of OTUs in colored and white biofilms after dividing subjects into healthy and severe caries groups. (c) Alpha diversity. Shannon index values for microbiomes in colored and white biofilms from the healthy and severe caries groups were calculated. Bars indicate standard error

**FIGURE 2:**
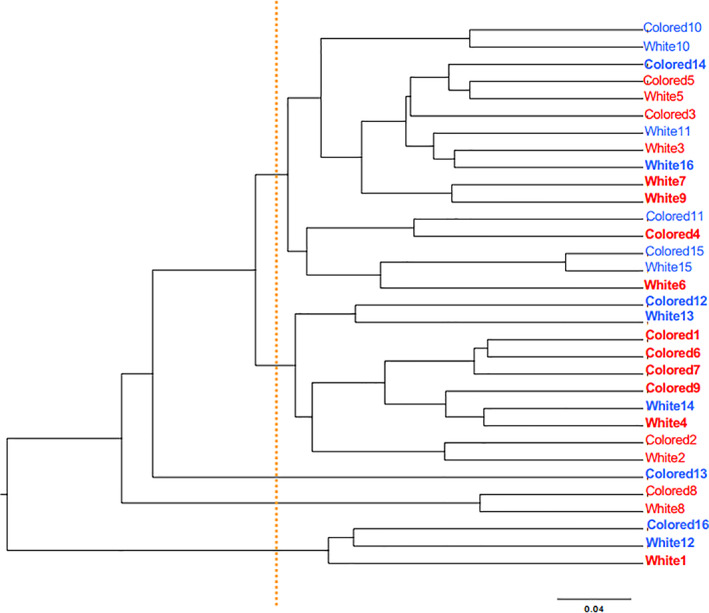
Phylogenic tree of colored and white biofilms. A phylogenic tree was constructed using results of weighted UniFrac distances, used as an index of beta diversity. Samples from the severe caries and healthy groups are shown in red and blue, respectively. Individual numbers (Subjects #1–9 in severe caries group, #10–16 in healthy group) have been added following the biofilm color description (colored or white). When the genetic distance between colored and white biofilms in the same subject was greater than the level indicated by the orange dotted line, the subject designation is shown in bold

The results indicated that the number of observed OTUs tended to be smaller for the colored as compared to white biofilms, though the difference was not statistically significant (Figure 1a). That number also tended to be higher in the severe caries than the healthy group, though again the difference was not statistically significant (Figure 1b). In addition, Shannon index, which shows microbial diversity in a community, was not significantly different between the groups (Figure 1c). Also, a phylogenic tree drawn on the basis of the weighted Unifrac distances did not show distinct patterns in either the colored or white biofilms (Figure 2), while PCoA plots indicated no phylogenetic differences between them (Figure S2). However, the phylogenic distance between colored and white biofilms was largely different, even in samples from the same subjects (#1, #4, #6, #7, #9, #12, #13, #14, #16; shown in bold in figure; Figure 2). This finding suggested that the bacterial composition differed in some subjects depending on biofilm color.

**FIGURE 3:**
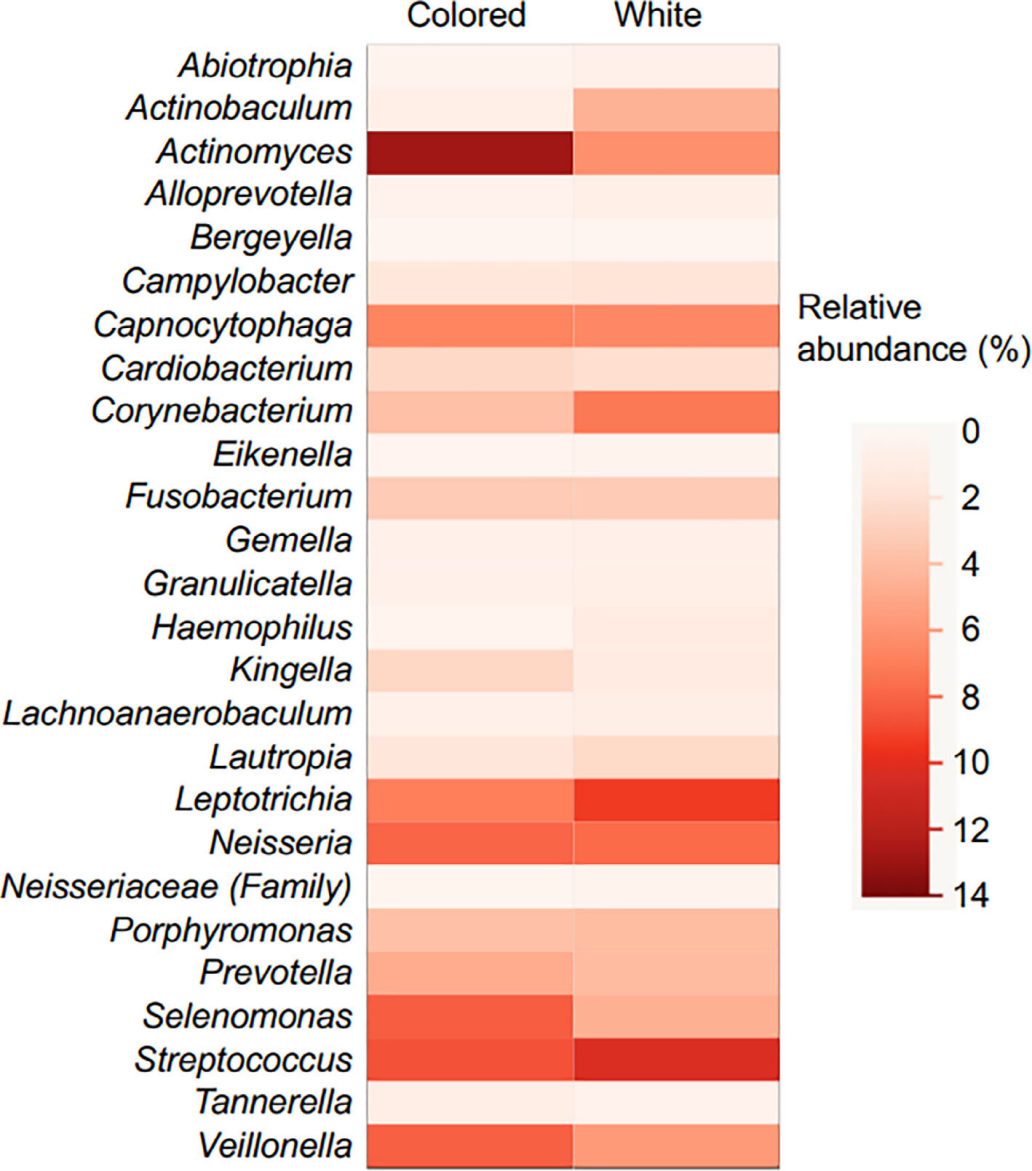
Heat map showing relative abundance of each bacterial taxon in colored and white biofilms. The average detection rate for each bacterial taxon in all reads among colored and white biofilms is schematically shown. Twenty‐six bacterial taxa, defined as core microbiomes for this study, were extracted and are presented

**FIGURE 4:**
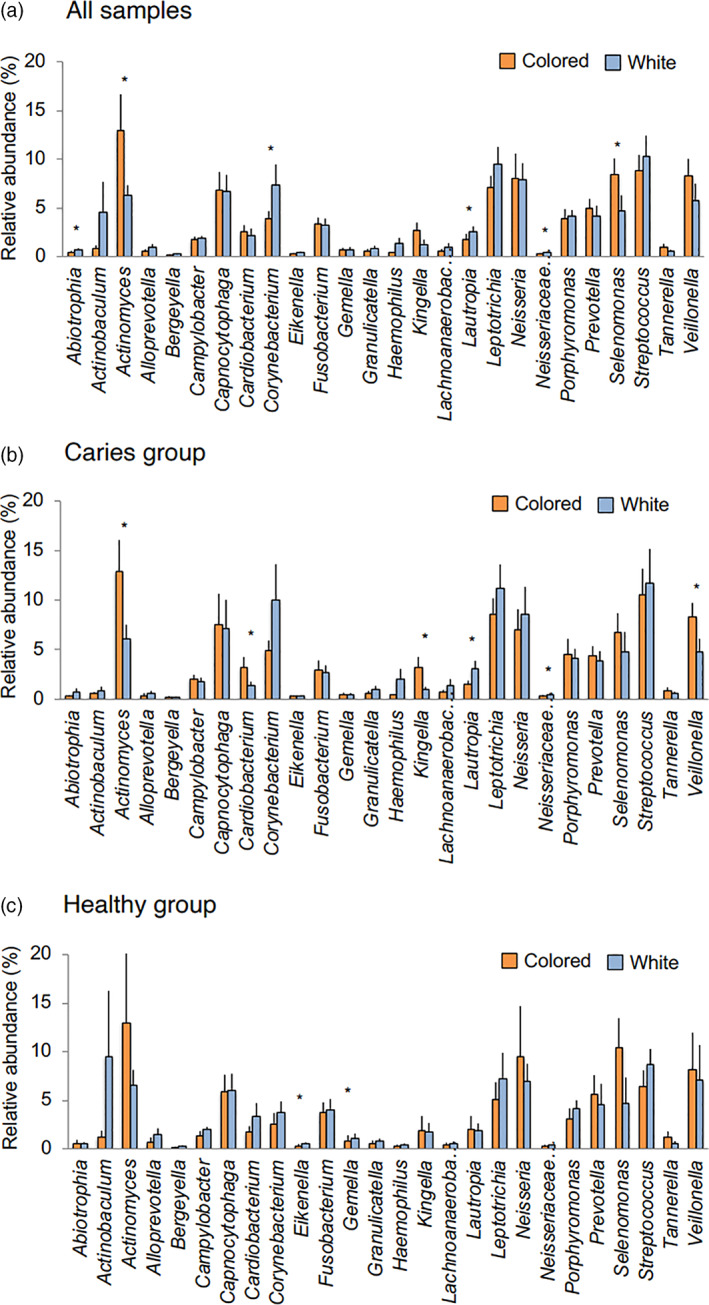
Relative abundance of 26 bacterial taxa in colored and white biofilms. The average detection rate for each bacterial taxon in all reads of colored and white biofilms is shown. Average value for (a) all samples, (b) samples from severe caries group, and (c) samples from healthy group. Bars indicate standard error. *p < .05, Wilcoxon's signed‐rank test

Of all estimated bacterial taxa at the genus level, 26 were detected in all samples and defined as core microbes for this study. A heat map was prepared for comparing the relative abundance of each taxon between colored and white biofilms. Those comparison results revealed marked differences in the abundance of some genera depending on biofilm color, with *Actinomyces*, *Selenomonas*, and *Veillonella* more frequently detected in colored biofilm (Figure 3). Furthermore, Wilcoxon's signed‐rank test results revealed that *Actinomyces* and *Selenomonas* were more frequently contained in colored biofilms, while *Abiotrophia*, *Corynebacterium* and *Lautropia*, and family *Neisseriaceae* were more frequently seen in white biofilms (Figure 4a). Next, relative abundance was compared between colored and white biofilms after dividing between the severe caries and healthy groups. Among samples obtained from the severe caries group, the *Actinomyces*, *Cardiobacterium*, *Kingella*, and *Veillonella* genera were detected at significantly higher frequencies in colored biofilms, while the frequency of *Lautropia* and family *Neisseriaceae* was significantly higher in white biofilms (Figure 4b). On the other hand, in white biofilms from the healthy group, only two genera, *Eikenella* and *Gemella*, were detected more frequently, though they comprised only a small percentage among all of the reads (Figure 4c). Among six taxa showing differences between colored and white biofilms in the severe caries group, two genera, *Actinomyces* and *Veillonella*, were observed with high frequency in all of the reads.

The genus *Actinomyces* taxon accounted for the highest percentage in the examined microbiomes. In particular, of 32 samples that underwent 16s rRNA gene pyrosequencing, the abundance of *Actinomyces* was higher than 15% of all reads in five samples and each of those were from colored biofilms (Table 2; *p* = .014, colored vs. white biofilm, Chi‐square test). Additionally, four of those five samples were obtained from subjects allocated to the severe caries group, though the difference as compared to the healthy group was not statistically significant (*p* = .197).

**TABLE 2:** Relative abundance of genus *Actinomyces*

	Number of samples	Number of samples >15%
All samples	32	5
Colored biofilms	16	5*
Severe caries group	9	4
Healthy group	7	1
White biofilms	16	0*

* *p* < .05, comparison between colored and white biofilms. Chi‐square test.

In adults, mature biofilm is known to cause dental caries and periodontal disease. However, in the present pyrosequencing results, nearly none of the reads were identified as known cariogenic or periodontopathic bacteria. Thus, we also attempted to detect bacteria species in colored and white biofilms from 32 subjects using PCR, though comparisons of the detection rate of each revealed no significant differences regardless of coloration (Figure S3a). Similarly, when that comparison was made separately with samples from the severe caries and healthy groups, no difference in detection rate associated with biofilm color was noted (Figure S3b).

**FIGURE 5:**
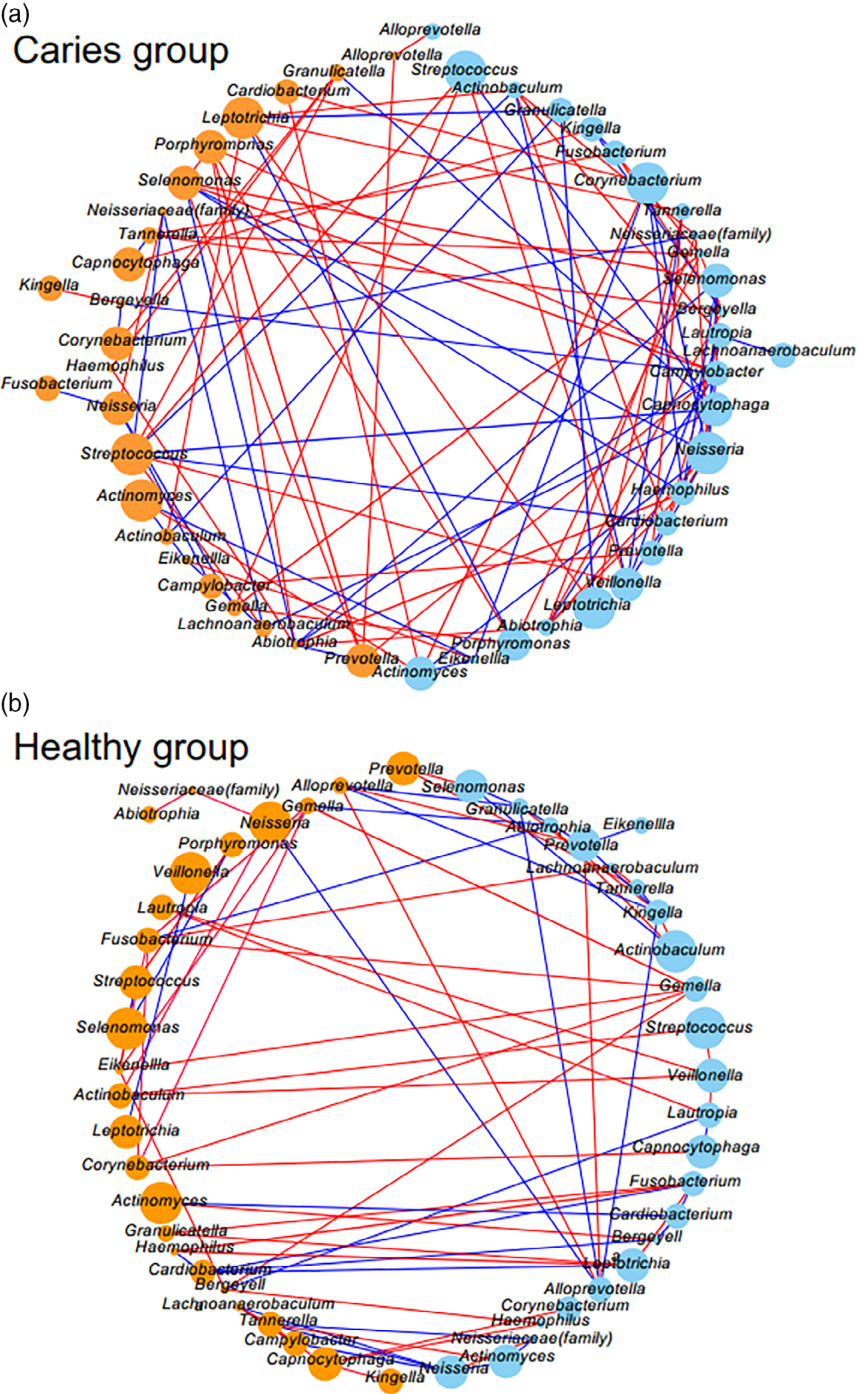
Co‐occurrence networks. (a) Severe caries and (b) healthy groups. Among the 26 bacterial taxa examined, all combinations with a positive (red) or negative (blue) correlation in terms of relative abundance are shown by lines. Thin lines show SCC between two taxa greater than 0.4 and thick lines show those greater than 0.85. Circle represents each bacterial taxon, and its size and color reflects relative abundance and biofilm color, respectively. Colored biofilms are shown as orange and white as light blue

To evaluate the influence of each of the 26 bacterial taxa included in the core microbiome on the abundance of other taxa, SCC values were calculated, with the results presented schematically to enable visualization (Figure 5). In the severe caries group, there were 108 combinations of taxa that showed positive or negative correlations in terms of bacterial abundance, while 80 such combinations were found in the healthy group. Taxa showing strong correlations with 10 or more other taxa in the severe caries group were *Abiotrophia*, *Campylobacter*, *Neisseria*, *Selenomonas*, and *Streptococcus*, of which 3 had a high relative abundance (>5%). In the healthy group, two taxa, *Fusobacterium* and *Gemella*, showed correlations with 10 or more taxa, though both genera occurred at a low percentage among all reads. It should also be noted that *Streptococcus* showed strong correlations with several other taxa in the severe caries group.

## 4 DISCUSSION

It is not uncommon to find colored biofilm on the tooth surfaces of children, though little is known regarding whether that is a type of mature biofilm caused by poor oral hygiene. Furthermore, dental practitioners are unable to determine whether such occurrence is associated with an elevated risk of oral disease, such as dental caries or periodontal disease. Since the mechanism of dental biofilm coloration in children is largely unknown, we examined microbiomes of obtained samples in the present study.

The dental caries status of the present subjects is shown in Table 1. Some children with colored biofilm had no dental decay or caries experience, suggesting that oral health status is not related to biofilm coloration. However, several of our subjects had severe dental decay or severe caries experience, thus we divided them into the severe caries and healthy groups, and conducted analyses.

The richness and diversity of the bacterial taxa tended to be slightly lower in the colored as compared to the white biofilm samples, though there was no significant difference for those features between the severe caries and healthy groups (Figure 1b,c). Colored biofilm seen in adults often undergoes maturation when not removed for a long period. Takeshita et al. analyzed microbiome changes due to biofilm maturation in adult subjects, and found gradual increases in observed OTUs (or bacterial taxa) and Shannon index values starting from the fourth day (Takeshita et al., 2015), while the characteristics of colored biofilm seen in the present child subjects were different. Among previous studies that analyzed the microbiome of dental biofilms obtained from patients with periodontal disease, several reported higher OTU and Shannon index values as compared with healthy control subjects (Abusleme et al., 2013; Griffen et al., 2011; Liu et al., 2012). Although a simplistic comparison of those results with the present is not possible, the results are not consistent. Examinations of periodontopathic bacteria using PCR conducted in the present study showed that detection rates did not differ between white and colored biofilms (Figure S3). Therefore, it is considered that colored biofilm occurring in children is not the same as mature biofilm seen in adults, thus it cannot be readily concluded that the risk of onset of dental caries or periodontal disease is high in children with biofilm coloration.

Phylogenetic tree and PCoA results revealed that the distance between colored and white biofilms was large in 9 of 16 of the present subjects (Figure 2), suggesting differences in bacterial composition (Figure S2; Griffen et al., 2011). However, even in those samples, no particular microbiome pattern was found regardless of biofilm coloration. It has been reported that the microbiome in dental biofilm varies depending on location (e.g., between incisors and molars, among buccal, lingual, and proximal surfaces; Shi et al., 2016; Zaura, Keijser, Huse, & Crielaard, 2009). In the present study, we examined microbiomes in both colored and white biofilm samples obtained from the same subjects. For that analysis, we collected white biofilm from an adjacent region of the same tooth, an adjacent tooth, or a tooth on the opposite side in order to avoid the influence of proximity on variations in bacterial composition. Interestingly, the characteristics (e.g., color strength, hardness, difficulty with removal) in some subjects differed between white and colored biofilm, thus additional evaluations are needed in the future to identify bacterial taxa that exert influence on the biofilm microbiome. Also, detailed analysis of the bacterial composition of colored biofilm samples may help to reveal the mechanism involved in coloration in children, which appears to differ from that for mature biofilm development occurring in adults.

There was a greater number of bacterial taxa at significantly different percentages between colored and white biofilm samples obtained from the severe caries group as compared to the healthy group. Notably, *Actinomyces* and *Veillonella* in the severe caries group had relatively high levels (Figure 4b). Thus, at least among the core microbiome, it is suggested that bacterial composition differences between colored and white biofilms are greater in children who are affected by severe caries. A previous study of biofilms from children aged 4–5 years shown as black staining in tooth cervical regions found those to be richer in *Actinomyces*, *Cardiobacterium*, *Haemophilus*, *Corynebacterium*, *Tannerella*, and *Treponema* genera as compared to children without such black staining (Li et al., 2015). Another study that used real‐time PCR assay results noted that biofilms from children with black staining of the teeth (mean age 7.9 years) contained a high level of *Actinomyces naeslundii* organisms, while both DMF index values and the percentage of children with caries experience were lower among those without black staining (Heinrich‐Weltzien, Bartsch, & Eick, 2014). In the present cohort, mean DMF and df index values were higher in children with biofilm coloration than in the corresponding age group reported by the Ministry of Health, Labour and Welfare of Japan (Survey of Dental Diseases, 2016), suggesting that their oral health status differed from that of children with black staining on teeth, likely because of the different bacterial compositions and characteristics between those conditions in children.

Comparisons of the relative abundance of *Actinomyces* species between biofilm specimens from patients with dental caries/periodontal disease and healthy control subjects have been reported, with dental biofilm or saliva from children and young adults with dental caries shown to have a greater abundance of *Actinomyces* species (Jiang, Gao, Jin, & Lo, 2016; Johansson, Witkowska, Kaveh, Lif Holgerson, & Tanner, 2016). On the other hand, a study of younger children who had not completed the deciduous dentition revealed more frequent detection of *Actinomyces* from subjects without dental caries (Holgerson, Öhman, Rönnlund, & Johansson, 2015). Furthermore, detection of *Actinomyces* in subgingival biofilm has been shown to be more frequent in healthy control subjects as compared to patients with periodontal disease (Abusleme et al., 2013; Liu et al., 2012; Tsai et al., 2018). However, *A*. *gerencseriae* and *A*. *massiliensis*, both of which belong to genus *Actinomyces*, were frequently detected in “supragingival” biofilm samples from patients with periodontal disease (Vielkind, Jentsch, Eschrich, Rodloff, & Stingu, 2015). Unfortunately, in vitro culturing of the *Actinomyces* genus is difficult to perform (Volante, Contucci, Fantoni, Ricci, & Galli, 2005), thus it remains unknown whether those bacterial organisms are pathogenic. Furthermore, consensus has not been reached regarding the function of *Actinomyces* in the microbiome of dental biofilm. In the present study, all five samples in which *Actinomyces* was observed at a percentage of 15% or greater were from colored biofilms (Table 2) and four of those were obtained from subjects in the severe caries group. However, the difference was not statistically significant because the number of samples containing a large amount of *Actinomyces* organisms was not adequate for analysis. Examinations of additional samples are needed to confirm this possibility.

Other than *Actinomyces*, *Veillonella* was also observed more frequently in colored than white biofilms in the severe caries group (Figure 4b). In several previous studies, *Veillonella* has been detected at a high frequency in biofilms from children with dental caries (Cephas et al., 2011; Cherkasov et al., 2019; Xu et al., 2014). In addition to those bacterial genera, *Streptococcus* species have been found in high abundance in dental biofilms obtained at the initial stage of formation (Dzidic et al., 2018; Heller et al., 2016). Biofilm is formed by aggregation with these bacteria, as well as colonization by other species using metabolites and extracellular products from these genera (Dzidic et al., 2018; Hojo, Nagaoka, Ohshima, & Maeda, 2009). Changes in composition and/or gene expression profiles of bacteria involved in the early stages of biofilm formation may have profound effects on biofilm structure.

In the present study, combinations of bacterial taxa with positive or negative correlations with each other were more numerous in the dental biofilm specimens collected from patients with severe caries as compared to those from healthy subjects, and some of those taxa were seen in great abundance (Figure 5). It is likely that both coordination and antagonism among multiple bacterial species are involved in a complex manner in formation of colored biofilm in the presence of severe caries, which results in a different oral environment as compared to that seen in a healthy individual. In particular, the *Streptococcus* genus showed strong correlations with several other taxa in the present severe caries group. In addition, we also observed combinations of two different taxa in biofilms with different coloration (Figure 5). Bacterial taxa that show a variety of combinations with other taxa from colored biofilm might be associated with alterations in color occurring because of changes in relative abundance. To further elucidate the pathogenicity of biofilm coloration, it will be necessary to examine not only bacterial composition but also perform functional analysis using another technique, such as metagenomic shotgun sequencing (Liu et al., 2012).

To summarize, in samples obtained from the present cohort, colored biofilms did not show a distinct pattern and no significant differences were noted as compared to white biofilms, though some cases had a bacterial composition that differed markedly between samples collected from the same subject. Furthermore, while there was no particular pattern for the differences between colored and white biofilms, some of those that showed coloration were rich with *Actinomyces* organisms, which seemed to be a characteristics of at least some of the colored biofilms. In addition, the abundance of the *Streptococcus* genus showed a strong correlation with several other taxa in our subjects with severe caries. Additional investigations regarding whether such changes in bacterial composition are associated with increased risk of oral disease will enable dentists to provide appropriate guidance for child patients with colored biofilm.

## 5 WHY THIS PAPER IS IMPORTANT TO PEDIATRIC DENTISTS

- Our findings may help pediatric dentists understand the characteristics of dental biofilm coloration in children. Differences in bacterial composition between colored and white biofilms are higher in children with a severe caries condition as compared to those without caries experience.
- In children with severe caries experience, dental biofilm coloration suggests an elevated risk of oral disease, while the clinical meaning of dental biofilm coloration may be different between children with and without severe caries experience.
- Presently, there are few reports on biofilm coloration, thus additional research findings are necessary show the significance of that condition.

## AUTHOR CONTRIBUTIONS

Nobuko Nagai and Hiromi Homma collected and analyzed the data; Atsuo Sakurai conceived the ideas; and Naoko Takahashi and Seikou Shintani led the writing.

## Supporting information


**Supplemental Figure S1 Representative case with colored biofilm obtained from first molar.** (A) Colored biofilm was observed on the buccal surface of an upper first molar (arrow) as well as a lower first molar (arrowhead). Locations other than the upper first and upper primary second molars were not investigated in this study. (B) Color scale bar used to judge presence of biofilm coloration. Biofilms matching the range shown by arrows were collected.

**Supplemental Figure S2 Principal coordinate analysis (PCoA) plots based on weighted UniFrac distance values.** Squares (severe caries group, #1–9) and circles (healthy group, #10–16) represent individual samples. Colored biofilms are shown as orange or light orange, and white biofilms as blue or light blue. (A) PC1 and PC2 plots. (B) PC2 and PC3 plots. (C) PC1 and PC3 plots. PC1, PC2, and PC3 components of PCoA comprised 34.10%, 17.01%, and 10.89%, respectively, of all bacterial community variations.

**Supplemental Figure S3 Detection rates of cariogenic and periodontopathic species using conventional PCR.** Thirty‐two children (12 in healthy group, 20 in severe caries group) were enrolled (Table 1). The percentages of subjects with positive bands shown by conventional PCR with specific primer pairs were calculated. (A) Detection rates for all colored and white biofilms. (B) Detection rates for colored and white biofilms after dividing subjects into healthy and severe caries groups.

**Supplementary Table S1** Bacteria detected by conventional PCR
**Supplementary Table S2**. Sequence reads after quality check.
