# Supplementary figures and images for: Microbiomes of colored dental biofilms in children with or without severe caries experience
Source: Clin Exp Dent Res. 2020 Aug 6;6(6):659–68. doi: 10.1002/cre2.317 (PMC7745070; doi:10.1002/cre2.317)

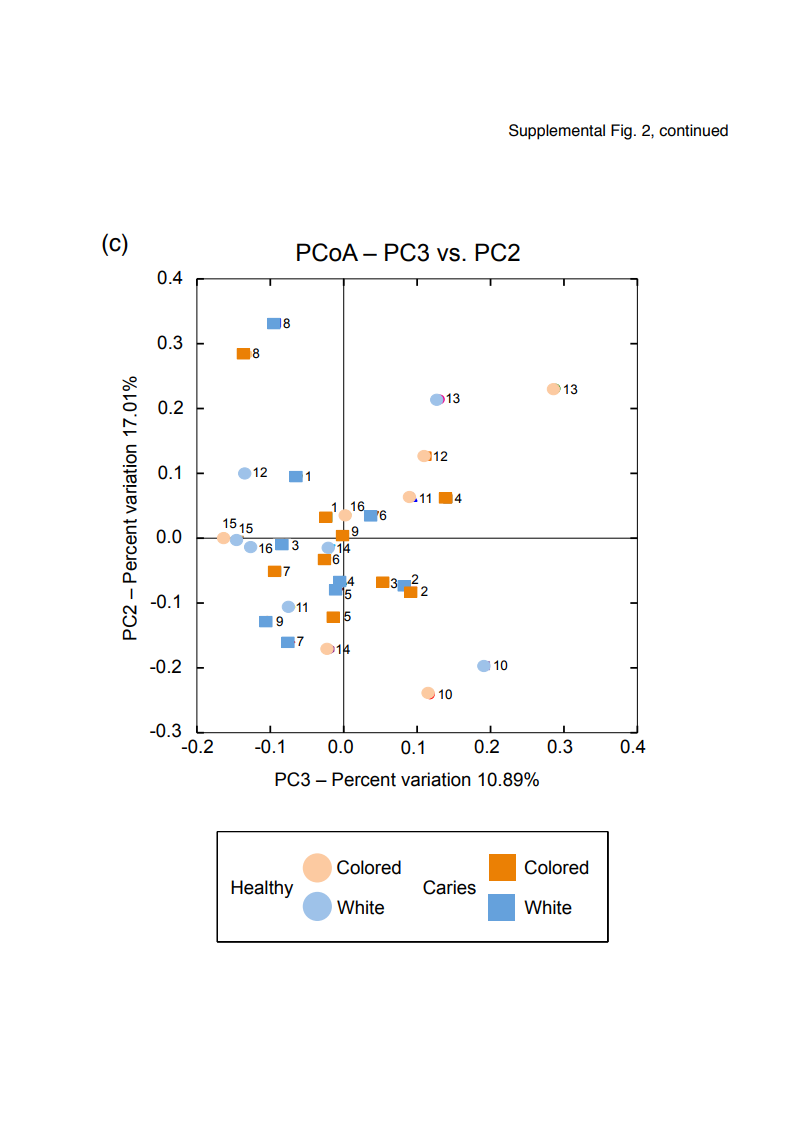

Supplement: Supplementary file 1 — Supplemental Figure S1 Representative case with colored biofilm obtained from first molar. (A) Colored biofilm was observed on the buccal surface of an upper first molar (arrow) as well as a lower first molar (arrowhead). Locations other than the upper first and upper primary second molars were not investigated in this study. (B) Color scale bar used to judge presence of biofilm coloration. Biofilms matching the range shown by arrows were collected. [file CRE2-6-659-s001.tiff]

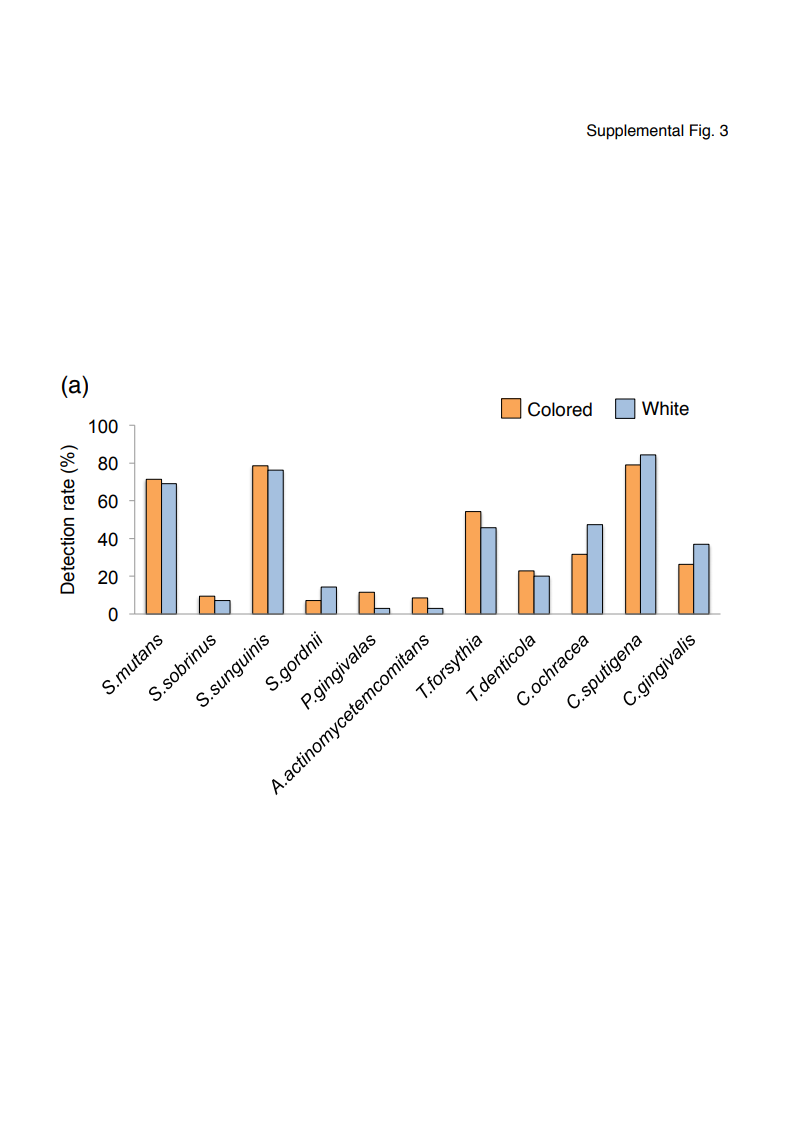

Supplement: Supplementary file 2 — Supplemental Figure S2 Principal coordinate analysis (PCoA) plots based on weighted UniFrac distance values. Squares (severe caries group, #1–9) and circles (healthy group, #10–16) represent individual samples. Colored biofilms are shown as orange or light orange, and white biofilms as blue or light blue. (A) PC1 and PC2 plots. (B) PC2 and PC3 plots. (C) PC1 and PC3 plots. PC1, PC2, and PC3 components of PCoA comprised 34.10%, 17.01%, and 10.89%, respectively, of all bacterial community variations. [file CRE2-6-659-s002.tiff]

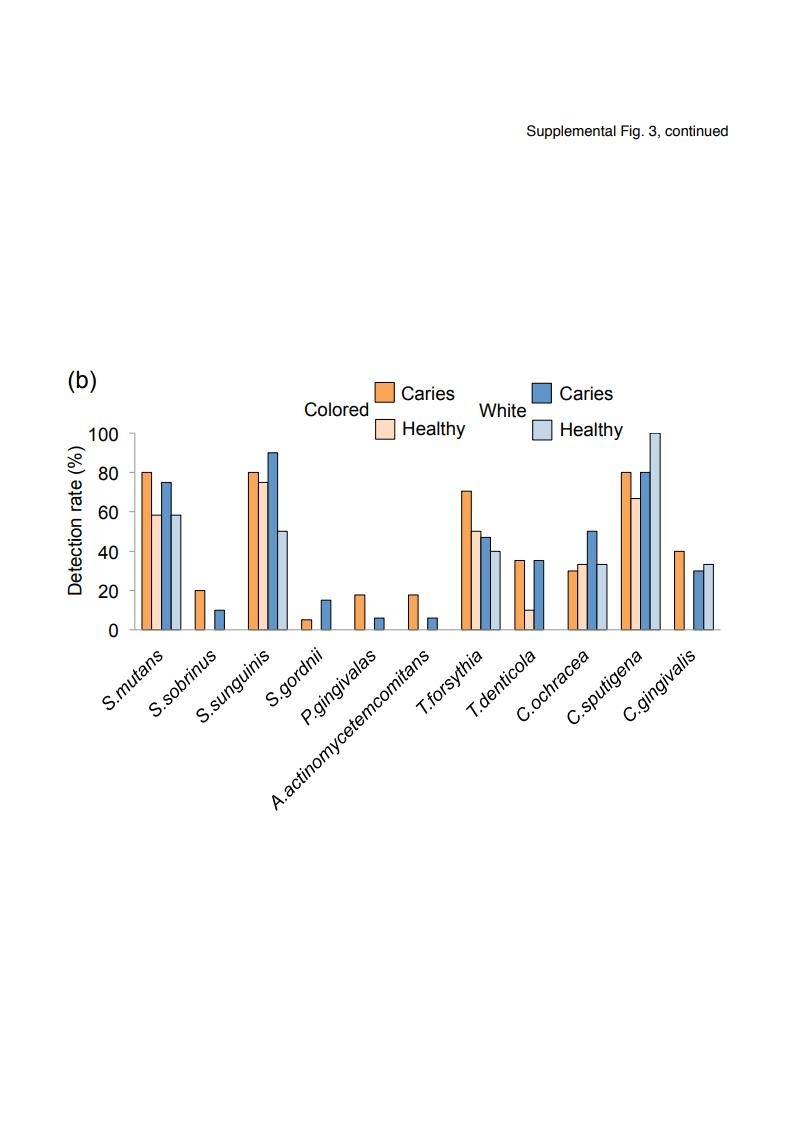

Supplement: Supplementary file 3 — Supplemental Figure S3 Detection rates of cariogenic and periodontopathic species using conventional PCR. Thirty‐two children (12 in healthy group, 20 in severe caries group) were enrolled (Table 1). The percentages of subjects with positive bands shown by conventional PCR with specific primer pairs were calculated. (A) Detection rates for all colored and white biofilms. (B) Detection rates for colored and white biofilms after dividing subjects into healthy and severe caries groups. [file CRE2-6-659-s003.tiff]
